# The Impact of Quality Assurance Initiatives and Workplace Policies and Procedures on HIV/AIDS-Related Stigma Experienced by Patients and Nurses in Regions with High Prevalence of HIV/AIDS

**DOI:** 10.1007/s10461-018-2066-9

**Published:** 2018-02-23

**Authors:** Sarah J. Hewko, Greta G. Cummings, Matthew Pietrosanu, Nancy Edwards

**Affiliations:** 1 Faculty of Nursing, Level 3, Edmonton Clinic Health Academy, University of Alberta, 11405 87 Avenue, Edmonton, AB T6G 1C9 Canada; 2 Faculty of Health Sciences, University of Ottawa, 1 Stewart Street, Room 205, Ottawa, ON K1N 6N5 Canada

**Keywords:** HIV, Social stigma, Africa South of the Sahara, Organizational policy, Quality assurance, Health care

## Abstract

Stigma is commonly experienced by people living with HIV/AIDS and by those providing care to HIV/AIDS patients. Few intervention studies have explored the impact of workplace policies and/or quality improvement on stigma. We examine the contribution of health care workplace policies, procedures and quality assurance initiatives, and self- and peer-assessed individual nurse practices, to nurse-reported HIV/AIDS-stigma practices toward patients living with HIV/AIDS and nurses in health care settings. Our sample of survey respondents (n = 1157) included managers (n = 392) and registered/enrolled nurses (n = 765) from 29 facilities in 4 countries (South Africa, Uganda, Jamaica, Kenya). This is one of the first studies in LMIC countries to use hierarchical linear modeling to examine the contributions of organizational and individual factors to HIV/AIDS stigma. Based on our results, we argue that organizational interventions explicitly targeting HIV/AIDS stigma are required to reduce the incidence, prevalence and morbidity of HIV/AIDS.

## Introduction

Despite the fact that 30 years have passed since the HIV virus was first identified, those diagnosed with HIV/AIDS continue to face significant discrimination and stigma [1]. Stigma is commonly experienced by people living with HIV/AIDS, those perceived to be HIV-positive [2, 3] and those who provide formal or informal care for HIV/AIDS patients. The latter is referred to as stigma by association [4–6]. It occurs when families, friends, neighbours, co-workers or patients fear that interacting with the health worker (regardless of his/her HIV/AIDS status) could lead to infection. Unfortunately, stigma by association creates significant barriers to health workers seeking prophylactic treatment in the case of a needle-stick injury, seeking early diagnosis, or adhering to treatment [7]. Stigma by association increases service users’ (i.e. patients, clients) self-stigmatization [6] and can adversely influence providers’ relationships [8], job satisfaction [6] and work-life quality [4]. Stigma is especially problematic—most notably as a strong barrier to accessing treatment—in low resource settings [9] where the HIV/AIDS prevalence rate is high and health human resources are limited.

Effectively reducing stigma is considered a critical intervention to halt the HIV/AIDS epidemic. However, most interventions tested in lower- or middle-income countries (LMICs) have focused on community approaches oriented toward individuals, such as educational, behavioural and cognitive strategies. In a recent systematic review [10] of interventions to reduce HIV-related discrimination and stigma, only 7.3% (3/41) of included studies intervened exclusively at the organizational level; five of the seven studies of multi-level interventions included interventions aimed at the organizational level.

Stigma-inducing policies may be engrained in the workplace. Based on this, the Global Business Coalition on HIV/AIDS formally encouraged companies without formal HIV/AIDS programmes to establish them [11]. Notable gaps in the study of workplace policies [12] and quality assurance efforts to address stigma exist. Following analysis of four feasibility studies of workplace interventions to lessen tuberculosis (TB) and HIV/AIDS stigma among health care workers, Siegel et al. [12] concluded that successful stigma-reduction campaigns need to address drivers of stigma at individual-, community- and structural-levels and employ iterative, participatory approaches.

Only ten of 48 studies included in Stangl et al.’s [10] review reported on interventions targeted towards health care workers; six aimed to increase knowledge of HIV/AIDS—its symptoms, transmission, and treatment—among service providers (nurses, physicians etc.), with the intention of both enhancing HIV/AIDS prevention and reducing stigma by service providers. Interventions in health care settings most often combined providing information at the individual-level with organizational skill-building activities, such as providing materials required for universal precautions and revising hospital policies. Stangl et al. [10] also highlighted the importance of developing, validating and employing tools that more precisely measure outcomes of stigma-reducing interventions. We would add that sophisticated methods of analysis—such as multi-level modelling—may enhance researchers’ ability to isolate the contributions of organizational contextual factors on outcomes at the level of individual health care providers and/or patients.

To date, few intervention studies have explored the impact of workplace policies and/or mandates for total quality improvement on stigma toward people living with HIV/AIDS and/or their health care providers. Feyissa et al. [13] conducted an observational study exploring health care provider stigmatization of people living with HIV/AIDS in Ethiopia and found that institutional support, as perceived by health care providers, predicted a reduction in stigma against people living with HIV/AIDS. The measure of perceived institutional support included supply-related, policy-related and protocol-related support. According to key informants in the study, no existing policies explicitly addressed either people living with HIV/AIDS or those providing their care. Although inconclusive with regards to the specific elements of policies and/or protocols that most effectively reduce health care provider’s stigma, these results indicate that policy and protocol as components of institutional support have the potential to reduce stigma [13].

A number of researchers have explored the role of quality assurance initiatives (distinct from workplace policies) on HIV/AIDS-related outcomes, such as mother-to-child transmission of HIV/AIDS [14, 15] and strengthening community-based health systems [16]. We were unable to find any studies of quality assurance initiatives that explicitly addressed HIV/AIDS stigma (toward either patients or care providers). Quality assurance initiatives are typically localized and dynamic (e.g. Webster et al. [17]); in the spirit of continuous improvement—quality assurance initiatives are often structured to accommodate multiple, iterative plan-do-study-act cycles [18]. In contrast, policies and procedures are typically developed and introduced synchronously across an organization in order to enhance the consistency of practices [19], both administrative and clinical. In general, policies and procedures (particularly administrative) are unlikely to be as consistently reviewed and modified as those implicated in quality assurance initiatives. Positive impacts of quality assurance initiatives have included improved protocols, strategic resource reallocation [15], improved reliability of complex, primary-care level treatment programmes [14], higher percent of patients retained in care, greater proportions of pregnant women agreeing to HIV/AIDS testing and operation of a greater number of antiretroviral therapy clubs [16].

In summary, few studies have explicitly tested the impact of organizational policies and procedures on HIV/AIDS stigma (toward either patients or health care providers). None that we could find studied the impact of quality assurance initiatives on HIV/AIDS stigma. The majority of interventions targeted individuals rather than organizations [10]; Stangl et al.’s [10] systematic review included 33 studies targeting individuals and only three with interventions at the level of the organization. We were unable to locate any study examining the combined contributions of quality assurance initiatives and policies in reducing stigma. Notably, both Stangl et al. [10] and Feyissa et al. [13] have concluded that individual-level interventions alone are inadequate to significantly reduce HIV/AIDS stigma. To date, researchers have not fully exploited the potential for multi-level analyses to highlight the relative contribution of each “level” (i.e. individual, organizational, structural) to stigma, as it is experienced by patients and/or front-line care providers.

### Study Purpose

The original study, a prospective quasi-experimental study evaluating the impact of newly-established leadership hubs on nurses’ care of adults living with HIV/AIDS in Jamaica, Kenya, South Africa and Uganda was conducted between 2008 and 2012; HIV/AIDS is highly prevalent in all of these countries. Data were collected pre- and post- establishment of leadership hubs. We direct readers to Edwards et al. [20] for a description of analyses conducted to answer the primary study question, which was: What are the impacts of establishing “multi-stakeholder leadership hubs on evidence-informed HIV care practices?” [20]. Unfortunately, results indicated that leadership hubs lacked the force to enhance uptake of evidence-informed practice in HIV care. The authors concluded that hub success is dependent on greater integration within health authorities; such integration facilitates regularization of hubs and would enhance hub sustainability [20]. In this paper, using the original post-intervention sample with participants from all four countries, we examine the contribution of organizational interventions, including health care workplace policies, procedures and quality assurance initiatives, and self- and peer-assessed individual nurse practices, to nurse-reported HIV/AIDS-stigma practices toward patients living with HIV/AIDS and nurses in health care settings. The study questions were as follows:

1. Does the presence of organizational-level HIV/AIDS-related workplace policies and procedures predict nurse-reported HIV/AIDS stigma toward nurses and patients?
2. Does the presence of quality assurance initiatives predict nurse-reported HIV/AIDS stigma toward nurses and patients?
3. Does the quality of individual level nursing practices predict nurse-reported HIV/AIDS stigma toward nurses and patients?

Our hypotheses were that, in the study countries: (1) the presence of organization-level HIV/AIDS-related workplace policies and procedures would predict fewer incidents of nurse-reported HIV/AIDS stigma toward nurses and patients; (2) the presence of quality assurance initiatives would predict fewer incidents of nurse-reported HIV/AIDS stigma toward nurses and patients, and; (3) a higher quality of individual level nursing practices (whether self-assessed or peer-assessed) would predict fewer incidents of nurse-reported HIV/AIDS stigma toward nurses and patients.

## Methods

### Sample and Setting

For a comprehensive description of the leadership hub intervention, refer to Edwards et al. [20]. This intervention was implemented in three districts/parishes of each participating country—Jamaica, Kenya, South Africa and Uganda. Three control districts/parishes were also selected in each country, except South Africa, where no control districts were sampled. There were some country-specific differences noted at baseline (see Edwards et al. [20] for details); for example, Ugandan participants were significantly less likely to report the existence of quality assurance initiatives and/or evidence-based HIV/AIDS policies and procedures than those in other countries. Notably, quality assurance initiatives were less commonly reported than evidence-based HIV/AIDS policies and procedures in all four countries [20]. All publicly-run health care institutions meeting World Health Organization (WHO) classification criteria for a level 3, 4 or 5 institution were eligible to be included in the study [21]. Facility size ranged from small, sub-district level health centres (level 3), which primarily provide primary health care services, to large, provincial or national referral hospitals (level 5) [20, 21]. Institutional classification criteria were consistent across countries.

A random, stratified sample of health institutions was selected in each district. Each national, provincial and parish/district hospital was provided with the protocol to randomly sample staff for inclusion in the study. All eligible staff employed in health centres were asked to participate; criteria for eligibility included fluency in English, employment in the health centre for a minimum of 3 months, profession of registered or enrolled nurse, and employment as a staff nurse or manager. Although institutions sampled were the same both pre- and post- intervention, individuals sampled pre-intervention were not necessarily the same as those sampled post-intervention. Only one wave of data was required to answer our secondary question; we elected to use post-intervention data because they were collected more recently. As noted by Edwards et al. [20], there was a general trend toward fewer reports of nurse stigmatization of patients in the study countries between baseline and follow-up. This trend was observed in both intervention and control groups [20].

### Data Collection and Measures

The baseline and follow-up questionnaires were identical. A complete description of the questionnaire is included in Edwards et al. [20]. For our analysis, we utilized post-intervention measures of clinical conduct, quality assurance initiatives, workplace policies and stigma. Measures of clinical conduct provided a self- and peer-assessment of the extent to which nurses or their peers consistently carried out evidence-based HIV/AIDS nursing practices. Measures of quality assurance and workplace policies examined nurses’ awareness of the existence of evidence-based quality assurance initiatives and of workplace policies in-line with evidence-based approaches to HIV/AIDS care. Measures were adapted from existing, validated instruments [22–24].

#### Individual Level Factors

Each nurse participant reported on: (1) their own clinical conduct (e.g. “*In my clinical care practice: I assess my patients’ comfort in disclosing his/her HIV/AIDS status to family members”*), and; (2) the clinical conduct of their coworkers (e.g. “*On my work unit: Nurses and midwives assess their patients’ comfort in disclosing his/her HIV/AIDS status to family members”*). Respondents were instructed to circle the response category indicating the frequency with which they (for the self-assessed clinical conduct scale) or other nurses and midwives on their unit (for the peer-assessed clinical conduct scale) provided the identified type of clinical care. The two clinical conduct scales each contained 12 parallel items measured via five-point Likert scale ranging from 1 (rarely) to 5 (always) [20].

#### Organizational Level Factors

The scales used to evaluate workplace quality assurance initiatives and workplace policies each contained 6-items with response options of yes, no and unsure. For example, one item in the quality assurance scale was “*At your workplace is there a quality assurance or quality improvement initiative in place to monitor the occurrence of occupational exposure to HIV/AIDS*?” One item in the workplace policy scale was: “*At your workplace, are there policies or procedures outlining the standard treatment of staff following exposure to HIV/AIDS in the workplace?*” [20]. Like Edwards et al. [20] we elected to code “unsure” responses as equivalent to a “no” response; if a manager is unaware of the existence of a quality assurance initiative or and/or workplace policy, then it is unlikely to significantly alter their practice or the practice of their employees.

#### Outcome Variable (HIV/AIDS Related Stigma)

We evaluated two distinct dimensions of HIV/AIDS-stigma that could be experienced in the health care system. The 10-item and 9-item scales respectively, evaluated stigmatizing experience in the past three months: the first evaluated stigmatization of people with HIV/AIDS by nurses (e.g. “*observing that a nurse kept her distance when talking to an HIV/AIDS patient*”), and; the second evaluated stigmatization of nurses providing care to people with HIV/AIDS by community members and nursing co-workers (e.g. “*observing someone say that nurses who care for HIV/AIDS patients spread the disease*”). A four-point scale used to capture responses ranged from 1 = never to 4 = most of the time; however, the post-intervention data revealed little variation in either of the sub-scale scores. This potential limitation of low variance has been addressed by previous authors [20, 25] in two ways—by creating a standardized mean score for each stigma sub-scale or by re-coding the responses to create a dichotomous variable, where 0 indicated never (original response = 1) and 1 indicated ever (original response = 2, 3 or 4) [20]. However, hierarchical linear modeling (HLM) requires a non-dichotomous variable for its outcome measure; at minimum ordinal, and if possible continuous [26, 27]. For this reason, we recoded the stigma sub-scales to create a single ordinal measure of stigma. Stigma scores are interpreted as: 0 = no observed stigmatizing events, either toward patients or nurses providing care; 1 = one stigmatizing event (either toward patients *or* nurses providing care) was observed; 2 = two or more stigmatizing events were observed, with at least one each toward patients *and* nurses providing care.

### Data Analysis

Research assistants in each country entered and cleaned data using Microsoft Excel™. For more detail on the original quantitative analysis of the data, refer to Edwards et al. [20]. Prior to conducting our analysis, we calculated correlations using the Spearman method. The data, as collected, incorporated questionnaire responses from both managers and nurses working with these managers. Therefore, the nested structure of the data, with managers at the facility (hospital) level and nurses within each hospital at the individual level, allowed us to use HLM [28]. We were not able to link the data from each nurse to a specific unit or manager; therefore, we developed a two-level model (nurses at level 1 and managers and facility characteristics at level 2), rather than a three-level model with nurses at level 1, managers/units at level 2 and facilities at level 3. Next, we determined theoretically which variables should be situated at each level (as per measures reported above) and then calculated Intra-Class Correlations (ICC2 s), to evaluate whether data showed that nurses were more alike in their responses within their facility than across facilities. Generally, a value of 0.70 or more indicates sufficient agreement across individual responses to warrant aggregation to the group/facility level [29]. Due to the nature of our question—exploring the impact of institution level initiatives on HIV/AIDS-stigma and the inherent nesting in the data collected—HLM was the best method of analyzing these data.

**Fig. 1:**
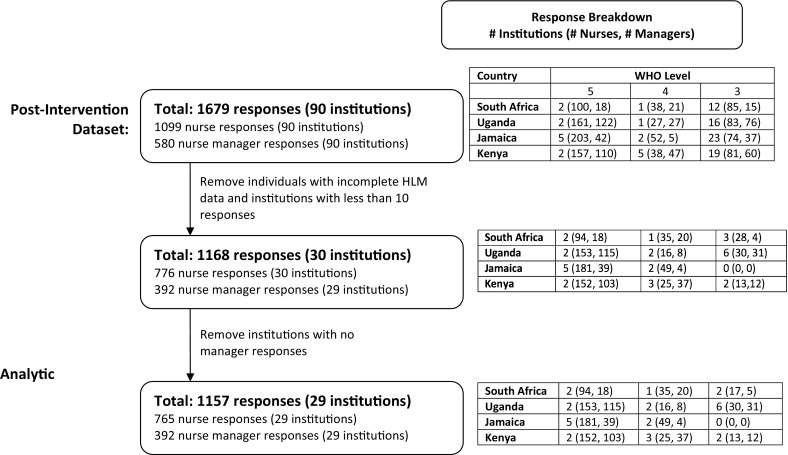
Analytic sample selection

To ensure that we had adequate power to conduct HLM we removed all institutions with fewer than 10 responses per institution. Fewer than 10 responses at an institution was not necessarily indicative of a poor response rate as sub-district and sub-parish health centres (Level 3 WHO Institutions) often had fewer than 10 nursing staff. This exclusion still provided the minimum sample of 25 facilities required at level 2 [30]. It is notable, however, that the number of Level 3 WHO institutions decreased to ten from 83 in the larger sample. Following this step, we reviewed the data to ensure that institution-level variables were defined for each included institution: as such we required a minimum of one manager response for each (see Fig. 1 for the analytic sample selection algorithm). Additionally, as HLM requires complete data (no missing values) for all variables of our model, we removed cases in which data were missing for any included variables. Thus, an observation was removed when a respondent did not answer one or more of the items in at least one of the survey sections. Scale values were derived as the average of all available survey items included in the scale (with reverse-coding as needed). See Fig.1 for details on the process of isolating the analytic sample, including 29 institutions and data from their managers and nurses.

We built the model step-wise by adding variables at level 1 first, then level 2, using model deviance statistic as a selection criterion. Predictor variables at the individual-level, utilizing responses from staff nurses, included self-assessment of clinical conduct (Clinical Conduct) and perceptions of coworkers’ clinical conduct (Co-Worker Clinical Conduct). At the facility level we included the measures of quality assurance initiatives (Quality Assurance) and workplace policies (Policies and Procedures) to determine whether having organizational workplace policies and procedures and quality assurance initiatives predicts HIV/AIDS-stigma behaviours toward nurses and patients. Manager responses within each organization were aggregated to the institutional level to represent the institution. Stigmatization—the outcome variable—was at the individual level, using data from staff nurses. Per this selection criteria, the variables described above were introduced into the model in the order of Clinical Conduct, Co-Worker Clinical Conduct, Policies and Procedures and Quality Assurance. The fitted models described, including a full univariate model that ignores the individual-facility hierarchical structure for comparison, are presented in Table 5.

Variables included in model estimations were considered as significantly contributing to prediction of the outcome variable when the *p* value in the regression output was less than 0.05.

## Results

### Sample Characteristics

**Fig. 2:**
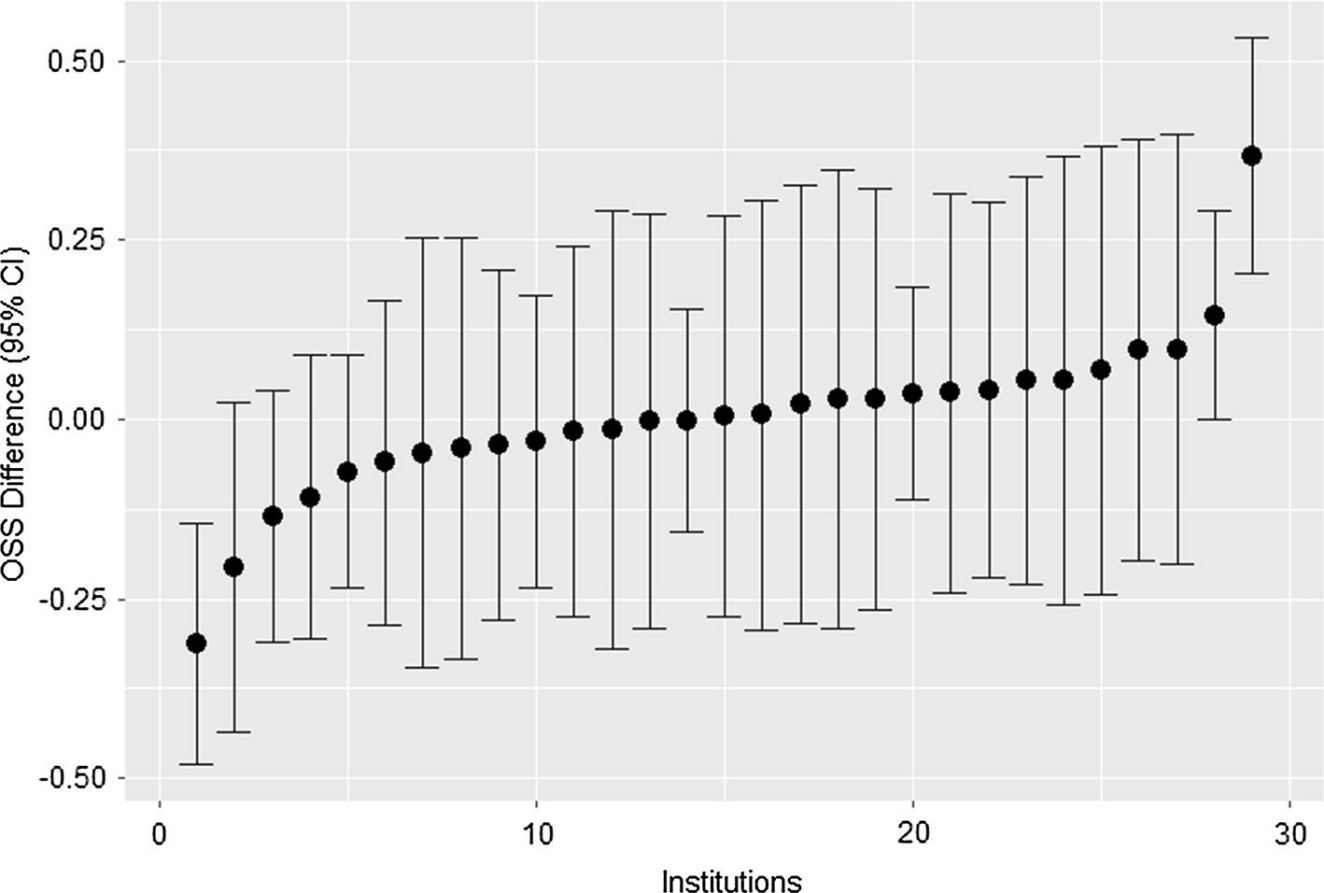
Inter-institution differences in average ordinal stigmatization score (OSS) in the 29 institutions included in the analytic sample. Mean OSS for each institution (in increasing order) is given by a single point, while the vertical bars around each point denote a 95% confidence interval for that institution. All values are with reference to—that is, centred about—the mean OSS for all institutions

The analytic sample (n = 1157) included managers (n = 392) and registered/enrolled nurses (n = 765) from 29 facilities—5 in South Africa, 10 in Uganda, 7 in Jamaica and 7 in Kenya. Ten of the facilities were health centers (Level 3 WHO Institutions), 8 (Level 4) were at the district/parish level and 11 were national/provincial (Level 5). Across the 29 facilities, a range of 1-88 and a median of 10 managers responded per institution. The managers in the sample were 89% female and 77% reported interacting with HIV/AIDS patients daily. Seventy percent of managers in the sample worked in national/provincial (Level 5) institutions, 18% in district/parish (Level 4) institutions and 12% in health centers (Level 3). The nurses in the sample were 93% female and 67% reported interacting with HIV/AIDS patients daily. See Table 1 and Fig. 1 for a description of the samples. See Table 2 for means, standard deviations (SDs), and ranges of scores for all included scales. No significant difference in scale means, SDs and ranges were seen between the full sample (including all participants) and those of the smaller, analytic sample (Table 3). Figure 2 visually displays inter-institution differences in average ordinal stigmatization score.

**Table 1:** Sample characteristics (n = 1157)

	Analytic sample (n = 1157)	Full sample (n = 1679)
Gender proportion		
Non-managers	F: 93% M: 6% No response: 1%	F: 92% M: 7% No response: 1%
Managers	F: 89% M: 10% No response: 1%	F: 89% M: 10% No response: 1%
Profession proportion		
Non-managers	Nurse: 23% Midwife: 52% No response: 25%	Nurse: 23% Midwife: 51% No response: 26%
Managers	Nurse: 4% Midwife: 65% No response: 31%	Nurse: 3% Midwife: 59% No response: 38%
Interaction with HIV/AIDS patients		
Non-managers	Daily: 67% Less often: 30% No response: 3%	Daily: 67% Less often: 30% No response: 3%
Managers	Daily: 77% Less often: 22% No response: 1%	Daily: 76% Less often: 23% No response: 1%

**Table 2:** Variable summaries for the analytic sample (n = 1157)

Variable	Note	Observed (theoretical) range	Mean	SD	ICC1
Clinical conduct (assessed by staff nurses)	For all nurse responses across all units	1.23–5.00 (1–5)	3.50	0.78	0.11
Co-worker clinical conduct (assessed by staff nurses)		1.31–5.00 (1–5)	3.73	0.77	0.10
Quality assurance (assessed by nurse managers)	Unit-level values used, with one value per unit (average response for all unit managers)	1.11–1.82 (1–2)	1.43	0.18	1.00^a^
Policies and procedures (assessed by nurse managers)		1.08–2.00 (1–2)	1.40	0.21	1.00^a^
Stigmatization	For all nurse responses across all units	0.00–2.00 (0–2)	1.14	0.80	0.05

These summaries apply to the variables relevant to the previous HLM models before grand-mean centering

^a^Necessarily 1 because scores were aggregated to the institutional level

**Table 3:** Comparison of full and analytic samples

	Analytic sample (n = 1157) Mean (SD)	Full sample (n = 1679) Mean (SD)
Clinical conduct (theoretical range 1–5)		
Non-managers	3.50 (0.78)	3.56 (0.81)
Managers	3.67 (0.70)	3.68 (0.67)
Co-worker clinical conduct (theoretical range 1–5)		
Non-managers	3.73 (0.77)	3.79 (0.77)
Managers	3.78 (0.67)	3.78 (0.66)
Quality assurance (theoretical range 1–2)		
Non-managers	1.40 (0.38)	1.37 (0.37)
Managers	1.43 (0.18)	1.38 (0.36)
Policies and procedures (theoretical range 1–2)		
Non-managers	1.40 (0.35)	1.35 (0.35)
Managers	1.40 (0.21)	1.35 (0.35)

We calculated an ICC2 of 0.60 for our ordinal measure of stigma, supporting its use as a level 1 variable. See Table 2 for ICC1s. Absolute values for correlations between variables included in the model ranged from 0.001 to 0.836. See Table 4 for the correlation matrix. These correlation measures indicate a significant positive correlation between mean scores for clinical conduct and co-worker clinical conduct and between mean scores for clinical conduct and stigmatization. Significant positive correlation was also seen between measures of quality assurance and policies and procedures and between measures of policies and procedures and stigmatization. These correlation measures indicated significant negative relationships between clinical conduct and quality assurance and between both quality assurance and policies and procedures with co-worker clinical conduct.

**Table 4:** Correlation matrix calculated using the analytic sample (n = 1157)

	Clinical conduct (assessed by nurses)	Co-worker clinical conduct (assessed by nurses)	Quality assurance (assessed by nurse managers)	Policies and procedures (assessed by nurse managers)	Stigmatization
Clinical conduct (assessed by nurses)		0.676***	− 0.098**	− 0.068	0.098***
Co-worker clinical Conduct (assessed by nurses)			− 0.145***	− 0.105**	0.001
Quality assurance (assessed by nurse managers)				0.836***	0.047
Policies and procedures (assessed by nurse managers)					0.107**
Stigmatization					

Significance levels: *0.05, **0.01, ***0.001

### Model Results

The model testing results are presented in Table 5. In the final model (Model 4), both clinical conduct and co-worker conduct significantly predicted stigmatization, with higher (better) self-assessed scores in clinical conduct significantly predicting higher (worse) stigmatization scores of people with HIV/AIDS, and higher (better) scores for perceptions of co-worker clinical conduct significantly predicting lower (better) stigmatization of people with HIV/AIDS. Despite not presenting as a significant predictor of stigmatization (p = 0.11), the addition of the institution-level measure of policies and procedures contributed 12% explained variance in stigmatization and, when added, significantly improved model fit.

**Table 5:** Results of multilevel analysis (of ordinal stigmatization score at two levels) using analytic sample (n = 1157)

Fixed effects	Univariate	Model 1	Model 2	Model 3	Model 4
Individual-level variables, coefficient (SE) (as assessed by nurses)					
Clinical Conduct	0.18 (0.05)***	0.09 (0.04)*	0.16 (0.05)**	0.16 (0.05)**	0.16 (0.05)***
Co-worker clinical conduct	− 0.12 (0.05)*		− 0.11 (0.05)*	− 0.11 (0.05)*	− 0.11 (0.05)*
Institution-level variables, coefficient (SE) (as assessed by nurse managers)					
Quality assurance	− 0.71 (0.34)*				− 0.42 (0.46)
Policies and Procedures	1.18 (0.35)***			0.40 (0.27)	0.71 (0.44)

Variance component	Null model variance	Regression models residual variance			
Institution level	0.033	0.031	0.031	0.027	0.026
Individual level	0.598	0.595	0.592	0.592	0.593
Total	0.637	0.626	0.623	0.619	0.619

Other statistics	ICC	Percentage (%) explained variance by regression models			
Institution level	0.05	6.4	8.3	20.0	23.1
Individual level	0.95	0.5	1.0	1.0	1.0
Model deviance	1802.4	1801.2	1800.6	1799.3	1798.2
Change in deviance from null	0	− 1.2	− 1.8	− 3.1	− 4.2

Significance levels: ‘***’ 0.001, ‘**’ 0.01, ‘*’ 0.05

## Discussion

To our knowledge, this is one of the first studies in LMIC countries to use HLM to analyze nested data, and to examine the contributions of organizational and individual factors to perceived stigma toward nurses caring for and patients affected by HIV/AIDS. Results reinforce the importance of using institutional policies to support destigmatizing approaches by nurses. Although other authors [31–34] have called for institutional policies to support clinical care and employee well-being, studies examining the relationship between the two are sparse. Through our analysis, we found that policies and procedures explained variance in the model and quality assurance measures did not. Although the items in the two measures were worded similarly, they described different types of intercession. As noted above, quality assurance initiatives are more likely to be localized, in that they may be exclusive to a single unit or program within a health organization; for this reason, knowledge of these initiatives among nursing staff may be less pervasive than knowledge of policies and procedures. It is possible that quality assurance initiatives made significant contributions to reduction of stigma in small pockets of the participant population but that these effects were diluted in the larger sample.

While this analysis focused on policies directed toward nursing care, human resource management (HRM) policies are also important as they influence health workers’ perceptions of how their rights and employment experiences will be safeguarded. While measures of HRM were included in the original surveys, these data did not meet the statistical requirements for an HRM analysis. Additional HRM findings from the study have been published in a separate manuscript [7].

Future research questions using HLM techniques could be significantly advanced by planning this approach from the outset of the study—prior to data collection. This could allow for partitioning of variance between the actions, characteristics and/or other factors at each individual level (nurse data), unit level (manager data and unit characteristics) and facility level (administrator data and organizational characteristics). However, this requires considerably larger samples sizes (with a minimum of 25 cases, preferably 30, at the highest organizational level) than may be required for more conventional analyses. More broadly, inclusion of measures of intercessions introduced by external bodies, such as governmental ministries responsible for health outcomes (e.g. media campaigns) or professional nursing associations (e.g. changes to professional licensing standards), may further add to our understanding of factors implicated in the reduction of HIV/AIDS stigma. In our view, the additional costs for studies designed to account for multiple avenues of influence on stigma are offset by the benefits of an advanced understanding of effective stigma reduction.

HLM also requires an adequate sample size within organizations and this presents another challenge. For a study similar to ours meeting this sample size requirement would exclude many of the smaller but most predominant health care institutions in LMICs. This is because staffing numbers in these peripheral health units are often minimal; and yet, the vast majority of health care services are delivered by health unit staff. Thus, to better understand facets of stigmatization at this important, peripheral level, it may be necessary to include all allied health staff, not just one particular professional group (in our case, nurses). In some of our earlier analysis, we found that stigmatization patterns reported by nurses in these peripheral health facilities differed from those of district and provincial hospitals. Surprisingly, reports of stigmatizing behaviours by nurses and their experience of stigmatization were lower in health units than in either district or provincial hospitals. This may reflect closer ties to the community of service and perhaps better knowledge of what policies exist. However, it seems unlikely to reflect stronger quality assurance approaches given the resource constraints in these settings. Had there been sufficient numbers of respondents at the health unit level, we may have found a significant interaction effect between stigmatization scores and the presence of organizational policies and procedures.

### Limitations

In our study, our analysis may have yielded more specific results if the tool used to evaluate HIV/AIDS stigma and discrimination had elicited more varied responses from study participants. Issues with measures of stigma have been reported by both Stangl et al. [10], who noted that existing scales have not been validated across multiple populations and contexts and Sengupta et al. [35], who concluded that many scales focus on assessing stigma in groups of uninfected individuals rather than in mixed populations (inclusive of infected and uninfected individuals). The stigma scale utilized in this study, however, was subjected to psychometric testing across five countries [25]. Stigma scores are often influenced by social desirability bias [36], which leads respondents to model their responses on cultural mores rather than on their beliefs and experiences. In this case, respondents may have considered a response at the lower end of the scale (i.e. never having observed stigma toward patients by nurses or stigma toward nurses) to be more socially desirable than a response at the high end of the scale (reflecting observed instances of stigma toward patients by nurses or toward nurses that occur “most of the time”). Social desirability is particularly troublesome in research designs that incorporate pre- and post-test assessments as participants may infer that researchers desire a reduction in the measure (in this case stigma) over time [36]. The lack of variance led us to merge the two dimensions of HIV/AIDS stigma (i.e. stigma by nurses against people with HIV/AIDS and stigmatization of nurses by co-workers and community members) and to collapse categories of frequency. Advancements in the field of stigma measurement may allow researchers to better differentiate between the relative effectiveness of workplace policies and quality assurance initiatives (and other interventions) to reduce stigma, as experienced by different targets.

As noted above, a limitation of HLM was the requirement for a minimum of 10 individual-level responses per institution; for this reason many institutions (with fewer than 10 individual-level responses) had to be removed from the analytic sample. Additionally, we were unable to directly connect an employee to their manager, which prevented us from conducting a more complex analysis that could have accounted for variance at three levels (staff, manager and facility). The role of manager likely varied across facilities—managers in WHO level three facilities would be more likely to be involved in direct clinical care while those in WHO level 5 facilities may have been directly involved in crafting policies and procedures and in designing and implementing quality assurance initiatives. In future studies, where feasible, information on reporting structure could be recorded. This would allow us to conduct analyses that could parse out the role of managers in reducing HIV/AIDS stigma.

## Conclusions

Stigma remains a barrier to optimal management of HIV/AIDS. The potential for organization-level interventions to reduce stigma, whether against patients living with HIV/AIDS or their health care providers (or both), has been under-explored. We argue that workplace policy and quality assurance initiatives explicitly targeted at HIV/AIDS stigma are required to reduce the incidence, prevalence and morbidity of HIV/AIDS and to realize the potential of innovation in HIV/AIDS diagnosis and treatment.
